# Trends in comorbidities and multimorbidity among adults with recently diagnosed diabetes: United States, 1999–2023

**DOI:** 10.1016/j.diabres.2026.113530

**Published:** 2026-08-27

**Authors:** Stephen Onufrak, Ibrahim Zaganjor, Daesung Choi, Sola Han, Christopher S. Holliday, Meda E. Pavkov

**Affiliations:** Division of Diabetes Translation, National Center for Chronic Disease Prevention and Health Promotion, Centers for Disease Control and Prevention, Atlanta, GA, USA

## Abstract

**Aims:**

Comorbidities are common at diabetes onset and may influence long-term outcomes. We examined national trends in the prevalence, number, and multimorbidity patterns among U.S. adults with newly ascertained diabetes, distinguishing concordant metabolic risk factors, concordant vascular diseases, and discordant conditions.

**Methods:**

We analyzed National Health and Nutrition Examination Survey data (1999–August 2023) for adults (≥20 years) with newly ascertained diabetes (diagnosed at the survey or ≤2 years prior; n = 4,243). Age-adjusted prevalence of 13 comorbidities, mean comorbidity count, and multimorbidity patterns were estimated across three periods. Trends were assessed using logistic and linear regression and pattern distributions using chi-square tests.

**Results:**

Obesity increased from 58.3% (1999–2006) to 71.0% (2015–2023) (p = 0.0003); Chronic lung disease rose from 15.4% to 22.8% (p = 0.007). Chronic kidney disease (~30%) and cardiovascular disease (11.8%–14.1%) were stable. Arthritis (~40%), sleep disorders (29.2%–38.5%), and dental caries (~30%) were common but stable. The mean comorbidity count increased from 4.25 to 4.51 (p = 0.038), with greater overlap of concordant and discordant conditions (p = 0.008).

**Conclusions:**

From 1999 to 2023, obesity, chronic lung disease, and total number of comorbidities increased among adults with new diabetes. Multimorbidity patterns increasingly encompassed both concordant and discordant conditions, potentially complicating management and worsening outcomes.

## 1. Introduction

Adults with type 2 diabetes have a high prevalence of both concordant and discordant comorbidities. Concordant comorbidities are conditions that share metabolic or vascular pathways with diabetes, including metabolic risk factors (e.g., obesity, hypertension, dyslipidemia, metabolic-associated steatotic liver disease (MASLD)) and vascular disease (e.g., cardiovascular disease, chronic kidney disease) [1–3]. Discordant comorbidities are conditions that do not share the same primary metabolic or vascular management pathways as diabetes, and therefore often require distinct clinical expertise and care strategies, even if some biological mechanisms may overlap; examples of discordant comorbidities include depression, dental caries, chronic lung disease, arthritis, and cancer [1–5]. Comorbid conditions increase health care utilization, costs, and self-care challenges [6–10], and together contribute to multimorbidity (having multiple comorbidities): a burden associated with higher mortality [8,11–13], poorer quality of life [14], and disparities in outcomes across racial and ethnic groups [15]. Emerging evidence suggests that combinations of concordant and discordant comorbidities may be especially harmful [8].

Although number of comorbidities present rises with diabetes duration [16–19], many adults present with multiple conditions at diagnosis [16–21]. With diabetes prevalence increasing in the United States [22], understanding multimorbidity patterns at onset is critical for anticipating prevention, management, and cost implications. Although recent work has examined cardiometabolic conditions among U.S. adults with new onset diabetes [21], less is known about trends in discordant comorbidities and their role in shaping multimorbidity. This study addresses that gap by examining national trends in concordant and discordant comorbidities, multimorbidity burden (number of comorbidities present), and comorbidity patterns among U.S. adults with recently diagnosed diabetes.

## 2. Materials and methods

Data from the 1999–2000 through August 2021–2023 cycles of the National Health and Nutrition Examination Survey (NHANES) were used to investigate the presence of 13 comorbidities among adults (≥20 y) with new diabetes. NHANES is a nationally representative, repeated, cross-sectional survey that measures the health of the U.S. population [22]. New diabetes was defined as either self-report of health care provider diagnosis within the previous 2 years [23] or hemoglobin A1c test result during NHANES examination of ≥6.5% and no self-report of previous diagnosis. A similar definition of new diabetes has been used in previous research [21]. From 1999 to August 2023, a total of 5,824 adults in the study sample had new diabetes. After excluding participants with missing variables, the final study sample contained 4,243 adults, of whom 2,419 (57.0%) had previously undiagnosed diabetes and 1,824 (43.0%) reported a diabetes diagnosis within the last 2 years.

*Comorbidities*We assessed 13 comorbidities classified within the concordant/discordant framework:

1. Concordant – Metabolic risk factors
  - Obesity: measured body mass index (BMI) ≥ 30 kg/m^2^.
  - Abdominal obesity: waist circumference ≥ 102 cm in men or ≥88 cm in women.
  - Hypertension: self-reported use of prescribed antihypertensive medication or a mean systolic blood pressure ≥ 130 mmHg and/or mean diastolic blood pressure ≥ 80 mmHg, calculated across all available blood pressure readings obtained during the study visit.
  - Dyslipidemia: self-report of prescribed cholesterol medication *or* high-density lipoprotein (HDL) ≤ 40 mg/dL or non-HDL ≥ 130 mg/dL.
  - Metabolic-Associated Steatotic Liver Disease (MASLD): hepatic steatosis index > 36 [24].
2. Concordant – Vascular disease
  - Chronic kidney disease (CKD): eGFR < 60 mL/min/1.73 m^2^ *or* urine albumin-to-creatinine ratio > 30 mg/g calculated using the 2021 CKD-EPI creatinine equation without race [25].
  - Cardiovascular disease (CVD): self-report of provider diagnosis of heart attack, coronary heart disease, angina, or stroke.
3. Discordant comorbidities
  - Arthritis: self-report of provider diagnosis.
  - Chronic lung disease: self-report of provider diagnosis of emphysema, chronic bronchitis, or asthma.
  - Untreated dental caries: NHANES oral examination findings.
  - Cancer: self-report of ever being diagnosed by a provider with any cancer other than non-melanoma skin cancer.
  - Depression: PHQ score ≥ 10.
  - Sleep disorders: self-report of having ever told a provider about sleep difficulties.

Among the 13 comorbidities considered in the prevalence analysis, nine were available during the entire study period and were included in the assessment of multimorbidity: CVD, hypertension, CKD, dyslipidemia, chronic respiratory disease, MASLD, history of cancer, arthritis, and obesity. Abdominal obesity was excluded because, like weight status as assessed by BMI, it is also a measure of adiposity.

Covariates were age (20–44, 45–64, 65–74, and ≥75 years), sex (male or female), educational attainment (<high school graduate, high school graduate, some college, or college graduate), race and ethnicity (non-Hispanic [NH] White, NH Black, Hispanic, and NH Other), and health insurance status (yes or no).

### 2.1. Statistical analysis

All analysis was completed with SAS 9.4 (Cary, NC) using procedures to account for the complex survey design and study weights of the NHANES survey. Mobile examination center (MEC) survey weights were used for analysis and were adjusted to account for use of multiple survey cycles [26]. To ensure stable point estimates with sufficient sample size, survey cycles were pooled to create three periods (1999–2006, 2007–2014, and 2015–March 2023). Trend analysis tests were conducted using unpooled survey cycles per National Center for Health Statistics *Guidelines for Analysis of Trends* [27].

Demographic characteristics across the three periods were compared using chi-square tests. Age-adjusted prevalence of each comorbidity was estimated using logistic models to estimate least square means for each of the three periods. For comorbidities where data were not available for every NHANES cycle (sleep disorders [available 2005–August 2023], dental caries [available 2001–2004 and 2009–March 2020], and depression [available 2005–August 2023]), separate survey weights were calculated to account for missing survey cycles and estimates for each of the three periods included only available cycles of data.

For multimorbidity, the number of comorbidities was compared categorically across the three periods using chi-square tests, where total number was classified as 0–2, 3–4, or 5–9 comorbidities based on previous literature [16,28]. The age-adjusted mean number of comorbidities was then calculated for each of the three periods using least square means in linear models. Additional models were then fit to assess multimorbidity trends within each demographic subgroup. In supplemental analysis, to examine the potential role of obesity in multimorbidity trends, the multimorbidity trend models were refit with obesity removed from the count outcome and added as a control variable in the model.

Finally, we compared the distribution of comorbidity pattern types across periods using a chi-square test. This paper used publicly available, de-identified data and was not subject to human subjects determination.

## 3. Results

Among adults with new diabetes, education and health insurance status differed significantly across study periods, but diagnosis status, age, sex, and race and ethnicity did not (Table 1). Mean HbA1c for adults with new diabetes was 7.21% overall and was significantly higher among those with previously undiagnosed diabetes (7.53%) than among those diagnosed within the previous two years (6.83%; p < 0.001; data not shown).

Concordant comorbidities remained highly prevalent across all three time periods. Metabolic-associated steatotic liver disease, abdominal obesity, hypertension, and dyslipidemia each affected over 80% of participants in each period and were stable across time (Table 2). Obesity rose significantly, from 58.3% in 1999–2006 to 71.0% in 2015–2023(p = 0.0003). Vascular disease prevalence was stable overall, with CKD affecting ~30% and CVD 11.8%–14.1%.

*Discordant comorbidities* were also common. Arthritis remained the most frequent, affecting ~40% in each time period. Sleep disorders affected 29.2–38.5% across periods. Untreated dental caries persisted at ~30%. Chronic lung disease rose significantly, from 15.4% to 22.8% (p = 0.007). Depression (10.6–11.9%) and cancer (9.2–11.1%) remained less frequent and stable.

*Multimorbidity* burden is shown in Fig. 1. The proportion of adults with new diabetes and five or more comorbidities was 38.1% in 1999–2006 and 45.9% in 2015–2023, while the proportion with two or fewer comorbidities was 14.0% in 1999–2006 and 9.3% in 2015–2023. However, these proportions did not differ significantly by period (p = 0.06). The age-adjusted mean number of comorbidities rose significantly (p = 0.038), from 4.25 (95% CI: 4.08–4.41) in 1999–2006 to 4.51 (95% CI: 4.36–4.66) in 2015–2023 (Table 3). In subgroup analyses, the mean number of comorbidities increased significantly for adults diagnosed in the last 2 years (4.22 to 4.60; p = 0.0228), females (4.41 to 4.76; p = 0.0385), non-Hispanic White adults (4.33 to 4.76; p = 0.0033), Hispanic adults (3.66 to 4.06; p = 0.0369), those with some college education (4.18 to 4.72; p = 0.0069), and insured adults (4.27 to 4.53; p = 0.0137). In supplemental analysis, when obesity was removed from the outcome and adjusted for in the model, trends in increasing comorbidity count became non-significant (p = 0.38; data not shown).

Multimorbidity patterns shifted significantly across periods (p = 0.03; Fig. 2) and was increasingly characterized by combinations of concordant and discordant conditions.

## 4. Discussion

This study documents the high prevalence of *concordant comorbidities*, including both metabolic risk factors and vascular disease, alongside discordant comorbidities among adults with new diabetes. The mean number of comorbidities present has risen in the past two decades across demographic groups, with multimorbidity patterns increasingly including discordant conditions.

Our findings build on prior research highlighting the prominence of cardiometabolic comorbidities at diabetes onset [21]. Regarding discordant comorbidities, we observed high prevalence of arthritis and sleep disorders—each affecting more than one-third of adults with new diabetes. While data on arthritis at diagnosis are limited, nearly half of adults with diabetes had arthritis in 2008–2010 [29]. Similarly, although our measure included all self-reported sleep disorders, not just sleep apnea, the prevalence we found is consistent with prior reports suggesting that up to 23% of adults with diabetes have sleep apnea [30]. We also identified untreated dental caries, a condition independently linked to mortality, in more than one-fourth of participants [31,32]. Chronic lung disease, including asthma and COPD, also increased, affecting over 20% of subjects by 2015–2023, which is concerning given its association with CVD and mortality [33]. Vascular disease prevalence remained substantial, with approximately 30% of adults having CKD and 12%–14% having CVD. Because more than half of participants had previously undiagnosed diabetes, some vascular complications may have developed before diabetes was recognized, potentially due to prolonged uncontrolled hyperglycemia, underscoring the importance of early detection and treatment of diabetes.

Multimorbidity was overwhelmingly common: by 2015–2023, ~90% of people with new diabetes had more than two comorbidities and nearly half had five or more. The mean number of comorbidities rose modestly but significantly from 4.25 in 1999–2006 to 4.51 in 2015–2023. This increase was not likely driven by increasing age, as the age distribution of the study population did not differ by time period and our analysis was age-adjusted. In supplemental analyses, when obesity was removed from the comorbidity count and models were additionally adjusted for obesity status, temporal trends in comorbidity count were no longer statistically significant. These findings suggest that rising obesity prevalence may have been a major contributor to the increasing multimorbidity burden observed over time. Obesity is strongly linked to multiple conditions, including hypertension, dyslipidemia, asthma, and osteoarthritis [34,35], and its increase may partly explain increases in respiratory and other obesity-related disorders [36]. Our results align with findings from the United Kingdom, where multimorbidity burden among adults with diabetes also increased between 2000 and 2019 [17]. Notably, in our study multimorbidity patterns increasingly combined concordant comorbidities (metabolic or vascular) with discordant comorbidities over time, echoing increases in some discordant comorbidities observed in the United Kingdom [17]. Other research from Israel found the co-occurrence of concordant and discordant comorbidities increased mortality 33-fold compared to no comorbidities among people with type 2 diabetes [8], underscoring the concerning trends we observed.

These patterns have important implications. Multimorbidity increases health care utilization, raising primary care, specialty care, and hospitalization rates 2 to 5 times compared to diabetes alone [8–10,37]. It also substantially increases costs: U.S. studies report a tenfold rise in health care expenditures among patients with ≥3–5 comorbidities versus none [28,38]. Beyond utilization and cost, multimorbidity increases mortality risk [12]. Although the increase in mean comorbidity count in this study was modest (4.25 to 4.51 conditions per person), even relatively small increases in multimorbidity burden at the U.S. population level may substantially increase demands on healthcare systems, complicate clinical management, and worsen health outcomes among adults with newly diagnosed diabetes. Since delaying comorbidity development reduces mortality among people with diabetes [39], the growing burden of comorbidities at diabetes onset may translate to worse outcomes in the future. Early intervention at or before diagnosis may represent a critical window for reducing long-term morbidity and mortality. Strategies to prevent or delay development of multimorbidity include intensive obesity prevention, early blood pressure and lipid management, smoking cessation, pulmonary health interventions, and integration of behavioral health screening at diabetes diagnosis.

This study has limitations. First, the small number of adults with new diabetes in each NHANES cycle limited our ability to detect smaller trends. Second, including participants diagnosed within 2 years may have slightly inflated prevalence estimates, as new comorbidities can manifest soon after diabetes develops. Although the inclusion of recently diagnosed and undiagnosed cases increased sample size and improved stability of estimates across survey cycles, the inclusion of individuals with undiagnosed diabetes could bias prevalence estimates downward for some self-reported comorbidities because undiagnosed individuals may have lower health care engagement and consequently lower rates of physician diagnosis of these conditions. Importantly, some relevant conditions such as retinopathy and neuropathy were not consistently available across the study population, and not all comorbidities were measured throughout the study period, limiting precision for some estimates and restricting which conditions could be included in multimorbidity analyses. In addition, measurement of discordant comorbidities relied largely on self-report, whereas many concordant metabolic conditions were assessed using standardized clinical measurements, which may have influenced prevalence comparisons. Discordant conditions may have been underascertained among individuals with less interaction with the healthcare system, including individuals with limited healthcare access and participants with previously undiagnosed diabetes. Consequently, prevalence estimates for some discordant comorbidities may have been underestimated relative to concordant conditions. In addition, because lipid-lowering medications are often prescribed for primary prevention in diabetes irrespective of lipid levels, our inclusion of medication use in the dyslipidemia definition may have overestimated the prevalence of clinical dyslipidemia. Finally, our study may have been underpowered to detect smaller changes in individual comorbidities. For example, despite large increases in obesity, we did not detect significant increases in CVD or arthritis, which are both associated with obesity.

In conclusion, the prevalence of obesity and chronic lung disease among adults with new diabetes increased from 1999 to 2023, alongside rising comorbidity burden and multimorbidity patterns involving discordant conditions. These developments may lead to higher health care utilization, costs, and premature mortality among U.S. adults with diabetes, highlighting the potential benefit of preventing or delaying development of comorbidities among people with, or at risk for, diabetes.

## Figures and Tables

**Fig. 1.**
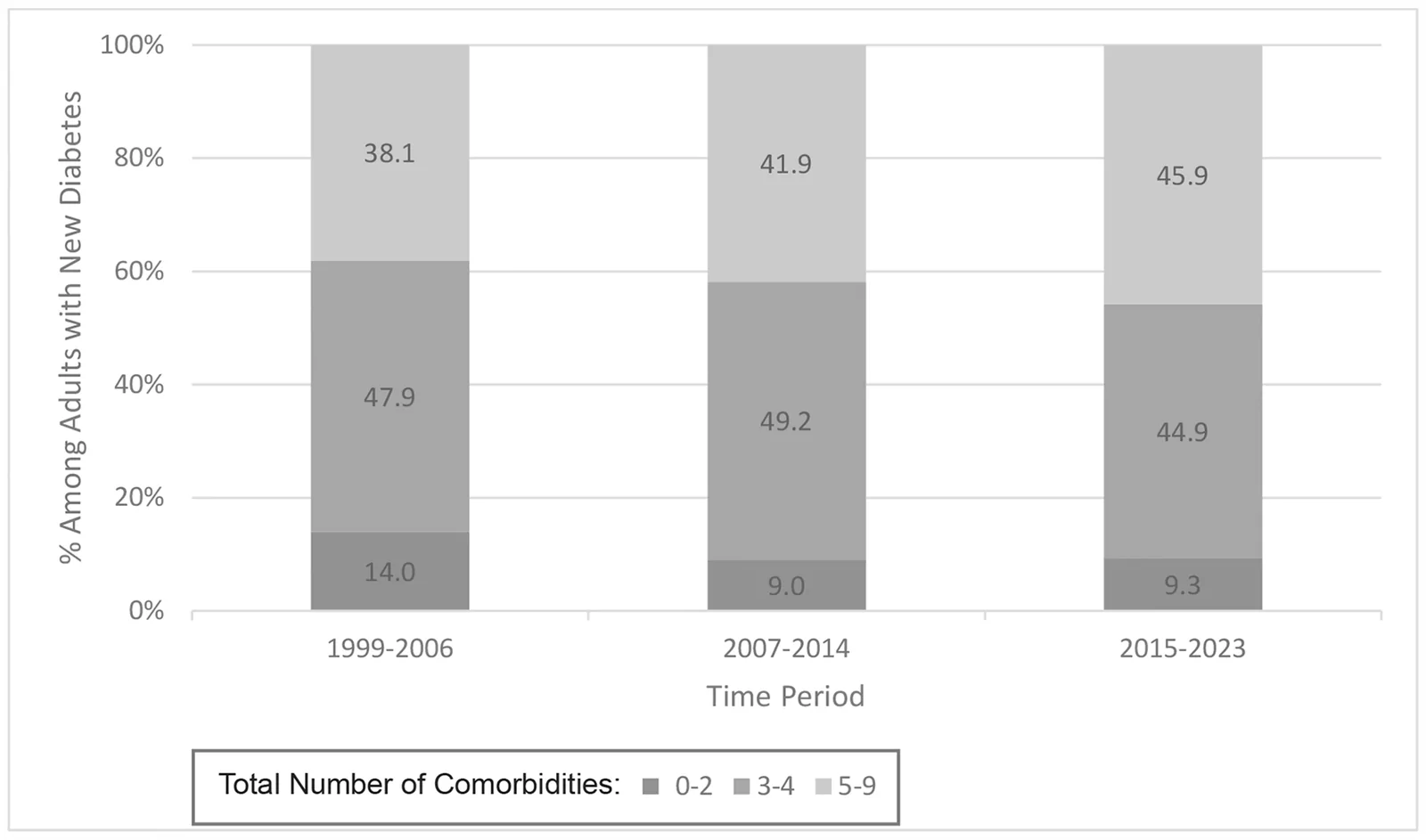
Total number of comorbidities present among adults with new diabetes across three time periods, National Health and Nutrition Examination Survey (NHANES), United States, 1999–August 2023. ^a^ Comorbidities assessed were cardiovascular disease, hypertension, chronic kidney disease, dyslipidemia, chronic respiratory disease, non-alcoholic fatty liver disease, history of cancer, arthritis, and obesity. ^b^ “New diabetes” defined as diagnosis during NHANES exam or self-report of doctor-diagnosed diabetes in the previous 2 years. Rao-Scott chi-square for differences in proportions by time period: p = 0.06.

**Fig. 2.**
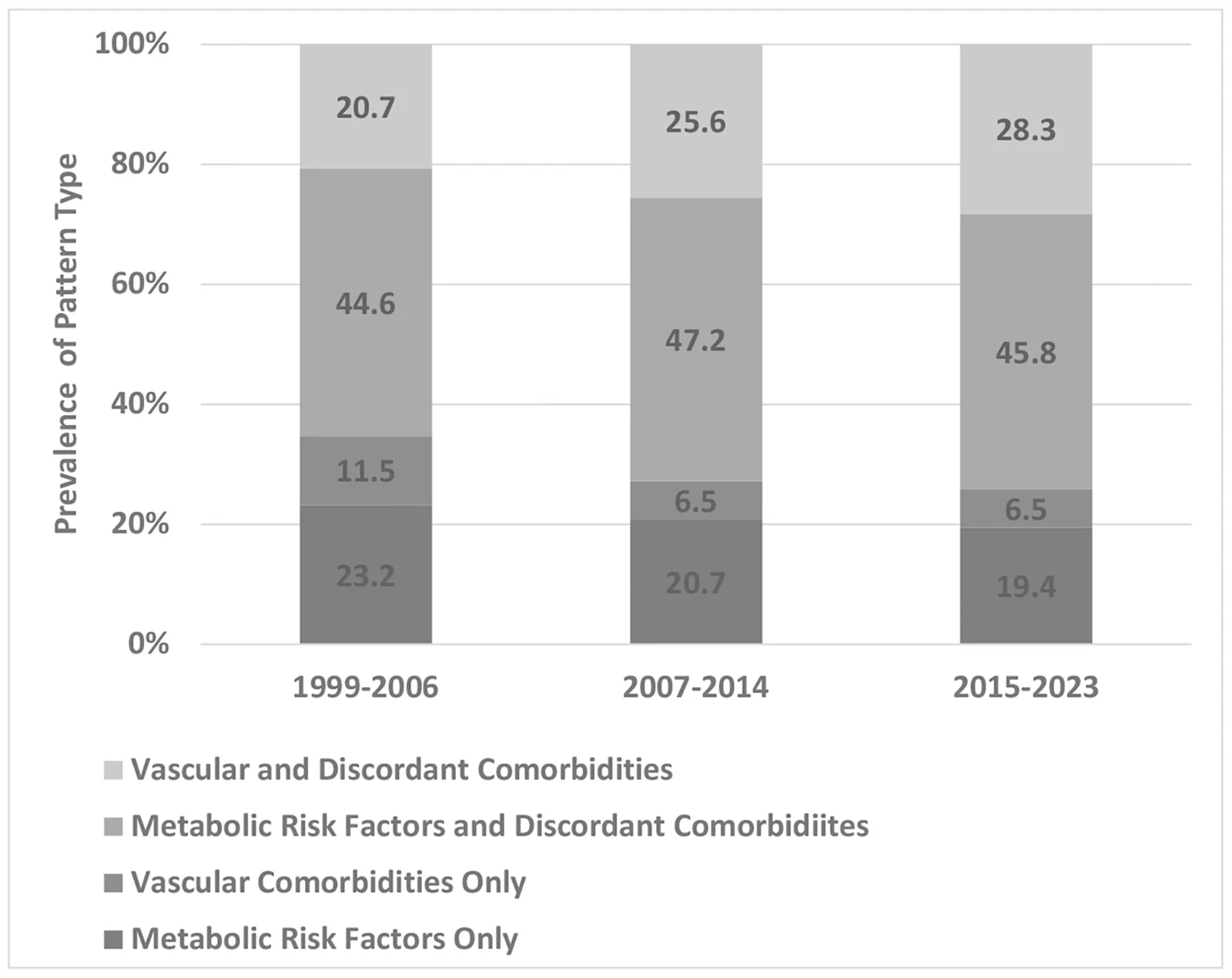
Prevalence of multimorbidity pattern types across time periods among adults with new diabetes, United States, 1999-August 2023. ^a^ Metabolic risk factors include hypertension, non-alcoholic fatty liver disease, dyslipidemia, and obesity; Vascular Disease includes cardiovascular disease and chronic kidney disease; Discordant Comorbidities include chronic respiratory disease, history of cancer, and arthritis. <1% of subjects within each time period had no comorbidities or only discordant comorbidities. ^b^ “New diabetes” defined as diagnosis during NHANES exam or self-report of doctor-diagnosed diabetes in the previous 2 years. Rao-Scott chi-square for differences in proportions by time period: p = 0.03.

**Table 1:** Characteristics of adults with new diabetes, national health and nutrition examination survey (NHANES), United States, 1999–2023.

Characteristic	1999–2006 (N = 824) Proportion (%)	1999–2006 95% CI	2007–2014 (N = 2412) Proportion (%)	2007–2014 95% CI	2015–2023 (N = 1801) Proportion (%)	2015–2023 95% CI	Chi-square p-value
**Diabetes diagnosis status**							0.36
Diagnosed in last 2 years (self-report)	50.5	43.8–57.2	45.2	40.5–49.9	47.0	42.7–51.2	
Diagnosed during NHANES exam	49.5	42.8–56.2	54.8	50.1–59.5	53.0	48.8–57.3	
**Age (years)**							0.83
20–44	23.6	18.5–28.7	19.9	16.2–23.5	22.8	17.6–27.9	
45–64	50.4	45.1–55.7	53.3	49.6–57.1	53.3	46.4–56.5	
65–74	15.5	11.9–19.1	15.7	13.1–18.4	16.2	12.6–19.9	
≥75	10.4	8.3–12.6	11.1	8.7–13.5	9.5	7.3–11.7	
**Sex**							0.29
Male	56.9	52.2–61.6	52.2	47.3–57.2	51.3	45.8–56.7	
Female	42.1	38.4–47.8	47.8	42.8–52.7	48.7	43.3–54.2	
**Race and ethnicity**							0.77
Non-Hispanic White	60.2	54.1–66.4	58.0	51.8–64.3	54.8	47.0–62.6	
Hispanic	15.7	10.9–20.5	16.9	12.6–21.3	20.0	15.2–24.9	
Non-Hispanic Black	15.6	11.9–19.4	17.1	13.2–20.9	15.2	10.9–19.5	
Non-Hispanic Other	8.4	4.8–12.1	8.0	5.4–10.6	10.1	7.2–12.9	
**Education**							
<High school	26.3	22.0–30.6	26.4	22.8–30.0	17.3	13.8–20.9	<0.001
High school	26.2	21.4–30.9	23.7	19.9–27.6	26.8	22.5–31.1	
Some college	29.7	24.2–35.1	34.5	29.7–39.3	31.2	26.5–36.0	
≥College graduate	17.9	13.8–21.9	15.5	12.4–18.5	24.6	19.6–29.6	
**Health insurance**							0.0017
Uninsured	14.7	11.9–17.5	20.3	16.3–24.3	12.3	9.3–15.2	
Insured	85.3	82.5–88.1	79.7	75.7–83.7	87.7	84.8–90.7	

**Table 2:** Age-adjusted time trends of comorbidities among adults with new diabetes^a^, National Health and Nutrition Examination Survey (NHANES), United States 1999–2023.

Comorbidity	1999–2006 Prevalence (%)	1999–2006 95% CI	2007–2014 Prevalence (%)	2007–2014 95% CI	2015–2023 Prevalence (%)	2015–2023 95% CI	p-value (Linear Trend)
**Metabolic Risk Factors**							
Metabolic-associated steatotic liver disease	87.4	84.0–90.1	90.4	87.1–92.9	89.0	85.7–91.7	0.29
Abdominal obesity	84.0	79.9–87.5	85.6	82.3–88.4	85.4	82.0–88.3	0.32
Hypertension	81.9	77.3–80.9	77.5	73.6–80.9	83.4	79.2–86.9	0.44
Dyslipidemia	91.6	87.6–94.3	89.1	85.6–91.8	89.6	85.6–92.6	0.19
Obesity	58.3	53.1–63.3	68.8	63.1–73.9	71.0	66.2–75.3	0.0003
**Vascular Disease**							
Cardiovascular disease	11.8	8.4–16.1	12.1	9.7–14.9	14.1	9.7–20.2	0.67
Chronic kidney disease	31.2	25.8–37.2	29.6	25.2–34.4	32.5	26.9–38.6	0.22
**Discordant comorbidities**							
Arthritis	39.2	33.6–45.0	40.6	35.8–45.6	41.6	37.0–46.4	0.34
Sleep disorder	35.1	24.6–47.4	29.2	24.7–34.1	38.5	31.1–46.3	0.14
Dental caries	33.0	26.8–40.0	28.3	23.1–34.2	30.0	23.4–37.6	0.56
Chronic lung disease	15.4	11.7–20.0	15.8	12.2–20.3	22.8	19.2–26.8	0.007
Depression	10.9	5.9–19.3	10.6	7.8–14.4	11.9	7.3–18.7	0.37
History of cancer	9.7	6.8–13.5	9.2	7.1–11.9	11.1	7.5–16.0	0.38

a “New diabetes” defined as diagnosis during NHANES exam or self-report of doctor-diagnosed diabetes in the previous 2 years.

b Sleep Disorder not available for 1999–2004 and 2021–2023; estimate for 1999–2006 based upon data from 2005 to 2006 (n = 318); estimate for 2015–2023 based upon data from 2015 to 2020 (n = 1180); trend test based on all available years.

c Dental Caries not available for 2005–2008 and 2021–2023; estimate for 1999–2006 based upon data from 1999 to 2004 (n = 767); estimate for 2007–2014 based upon data from 2009 to 2014 (n = 1280); estimate for 2015–2023 based upon data from 2015 to 2020 (n = 1180); trend test based on all available years.

d Depression not available for 1999–2004; estimate for 1999–2006 based upon data from 2005 to 2006 (n = 318); trend test based on all available years.

**Table 3:** Mean Number^a^ of Comorbidities^b^ Among People With New Diabetes^c^, by Time Period and Demographic Characteristic, National Health and Nutrition Examination Survey (NHANES), United States, 1999–August 2023.

Characteristic	1999–2006 Mean	1999–2006 95% CI	2007–2014 Mean	2007–2014 95% CI	2015–2023 Mean	2015–2023 95% CI	Annual Change	Linear Trend p-value
**Overall**	4.25	4.08–4.41	4.32	4.15–4.50	4.51	4.36–4.66	0.017	0.038
**Diabetes diagnosis status**								
Diagnosed in last 2 years (self-report)	4.22	3.99–4.44	4.49	4.24–4.73	4.60	4.30–4.89	0.026	0.0228
Diagnosed during NHANES exam	4.28	4.06–4.50	4.21	4.05–4.36	4.44	4.25–4.62	0.011	0.1581
**Age (years)**								
20–44	3.77	3.37–4.16	3.69	3.40–3.99	4.09	3.87–4.32	0.021	0.1356
45–64	4.14	3.90–4.38	4.19	4.02–4.35	4.45	4.20–4.70	0.019	0.0707
65–74	4.44	3.98–4.90	4.74	4.45–5.02	4.48	4.17–4.79	0.011	0.4488
≥75	4.58	4.17–4.99	4.84	4.46–5.22	4.85	4.44–5.26	0.018	0.2983
**Sex**								
Male	4.11	3.94–4.29	4.17	3.99–4.36	4.26	4.04–4.49	0.013	0.1048
Female	4.41	4.16–4.67	4.49	4.31–4.67	4.76	4.52–5.00	0.022	0.0385
**Race and ethnicity**								
Non-Hispanic White	4.33	4.13–4.54	4.51	4.30–4.73	4.76	4.51–5.02	0.029	0.0033
Hispanic	3.66	3.33–3.98	3.98	3.77–4.20	4.06	3.79–4.33	0.025	0.0369
Non-Hispanic Black	4.49	4.24–4.73	4.35	4.17–4.53	4.42	4.21–4.63	−0.001	0.8829
Non-Hispanic Other	4.06	3.45–4.67	3.48	3.11–3.85	3.96	3.55–4.37	−0.002	0.9318
**Education**								
<High school	4.33	4.02–4.65	4.47	4.22–4.72	4.14	3.91–4.37	−0.004	0.7632
High school	4.55	4.26–4.85	4.30	4.09–4.50	4.89	4.48–5.30	0.023	0.1166
Some college	4.18	3.87–4.49	4.43	4.21–4.65	4.72	4.53–4.91	0.031	0.0069
≥College graduate	3.78	3.44–4.11	3.90	3.52–4.29	4.08	3.82–4.35	0.025	0.0631
**Health insurance**								
Uninsured	4.06	3.74–4.39	4.07	3.86–4.28	4.28	3.77–4.79	0.016	0.3482
Insured	4.27	4.10–4.43	4.38	4.21–4.56	4.53	4.37–4.70	0.018	0.0137

a Age-adjusted.

b Comorbidities assessed were cardiovascular disease, hypertension, chronic kidney disease, dyslipidemia, chronic respiratory disease, metabolic-associated steatotic liver disease, history of cancer, arthritis, and obesity.

c “New diabetes” defined as diagnosis during NHANES exam or self-report of doctor-diagnosed diabetes in the previous 2 years.

## Data Availability

NHANES data are publicly available from the National Center for Health Statistics: https://wwwn.cdc.gov/nchs/nhanes/.
